# Directed Self-Assembly of Ge Quantum Dots Using Focused Si^2+^ Ion Beam Patterning

**DOI:** 10.1038/s41598-018-27512-z

**Published:** 2018-06-19

**Authors:** See Wee Chee, Martin Kammler, Jeremy Graham, Lynne Gignac, Mark C. Reuter, Robert Hull, Frances M. Ross

**Affiliations:** 1 Department of Materials Science and Engineering, Rensselaer Polytechnic Institute, Troy, NY 12180 USA; 2 Institut für Experimentelle Physik, Universität Duisburg-Essen, 47048 Duisburg, Germany; 3 Department of Materials Science and Engineering, University of Virginia, Charlottesville, VA 22904 USA; 4 IBM T. J. Watson Research Center, Yorktown Heights, NY, 10598 USA; 5 Present Address: Center for BioImaging Sciences, Department of Biological Sciences, National University of Singapore, Singapore, 117557 Singapore; 6 Present Address: Department of General Studies and Microsystems Engineering, Ostbayerische Technische Hochschule Regensburg, 93049 Regensburg, Germany; 7 Present Address: Thermo Fisher Scientific, Materials & Structural Analysis Division, Hillsboro, OR 97124 USA

## Abstract

We show that templating a Si surface with a focused beam of Si^2+^ or Si^+^ ions can create suitable nucleation sites for the subsequent growth of self-assembled Ge quantum dots by chemical vapor deposition. To determine the mechanism of patterning we use atomic force microscopy to show that, similar to Ga^+^ patterning, the formation of a surface pit is required to enable control over Ge quantum dot locations. We find that relatively high implantation doses are required to achieve patterning, and these doses lead to amorphization of the substrate. We assess the degree to which the substrate crystallinity can be recovered by subsequent processing. Using *in situ* transmission electron microscopy heating experiments we find that recrystallization is possible at the growth temperature of the Ge quantum dots, but defects remain that follow the pattern of the initial implantation. We discuss the formation mechanism of the defects and the benefits of using Si ions for patterning both defects and quantum dots on Si substrates.

## Introduction

The ability to arrange self-assembled semiconductor nanostructures such as quantum dots into arbitrary arrays or patterns is crucial for applications in nanoelectronics^1^ and optoelectronics^2^. One strategy for controlling nanostructure location is to create topological features on the substrate surface at which nanostructures will preferentially nucleate during subsequent growth^3–5^. Direct milling with focused ion beams (FIB) is a promising method for such surface templating^6^. The conditions for templating can range from high implanted doses, which mill deep features^7–9^ into the substrate, to low implanted doses, which either generate subtle changes in the surface topography on the order of a few nanometers or create point defects near the surface^10–14^. Low-dose templating^10–14^ is beneficial for patterning quantum dot arrays on planar surfaces because it does not significantly alter the substrate surface, leaving the substrate suitable for further processing. FIB-templated quantum dots have successfully been positioned within 100 nanometers of each another^8,13^, and recently, more complicated protocols have been attempted to reduce the separation between quantum dots even further^15^. The ultimate objective is to achieve precise control over the placement of quantum dots that also have well-defined physical structure, electronic structure, and doping. This will enable the fabrication of unconventional electronic logic devices that show promise for future electronics, such as the quantum cellular automata^16^, which require quantum dots to be within tens of nanometers of each other.

Ga^+^ is the most common ion species used for FIB templating due to its widespread availability in commercial FIB systems as the liquid metal source. Ga^+^ FIB is an effective tool to form patterns of individual Ge quantum dots on Si(001)^7–9^. This is achieved through both chemical and structural effects: pits induced by sputtering control the nucleation sites for Ge quantum dots, whereas the presence of Ga on the substrate surface modifies the growth kinetics of the Ge quantum dots^12,17^. However, Ga is a p-type dopant in silicon and it can become activated during the heat treatment required for re-crystallization^18^. Hence, its use is not ideal for electronic applications. The demonstration of patterns achieved using non-doping ions would be useful in extending the applications of FIB templating in electronic device fabrication.

A focused beam made up of other ions can be generated with a mass-separating FIB column (MS-FIB). MS-FIB differs from the conventional FIB column in that it uses a liquid metal alloy ion source containing the desired elements and integrates a Wien (or E × B) filter to separate the different ion species produced^19^. MS-FIB columns have been available since the early days of FIB development^20^ and ion beams of ~40 elements have been demonstrated in the literature using specific alloy sources^19,21,22^. The use of MS-FIBs has been explored for several applications in nanotechnology, including the selective doping of individual quantum dots^1^ and the creation of semiconductor quantum dot arrays^23^ by using the FIB to pattern lines into a thin layer of Si or Ge, followed by thermal de-wetting of the films. Au ion beams have been used to form catalyst particles for nanowire growth^24,25^ (Au^+^ in ref.^24^ and Au^2+^ in ref.^25^) and Ge^2+^ ion beams have been used to fabricate core-shell nanowire arrays^26^. MS-FIB has also been used for sample milling^27–29^ in situations where the presence of residual Ga is not welcome. Beyond these applications, other exciting opportunities remain to be explored for MS-FIB, especially in terms of nanoscale chemical modification.

In this paper, we show that the position of individual Ge quantum dots grown by chemical vapor deposition (CVD) at 600 °C can indeed be controlled by templating the substrate with a focused Si ion beam. We evaluate the process of Si ion implantation followed by Ge growth in an integrated vacuum system^30^, thereby avoiding complications from air exposure prior to Ge growth or the need to clean the substrate after patterning. Previously, the effects of Si^2+^ and Ge^2+^ ion implantation on the formation of SiGe “quantum dot molecules” have been examined (a process involving patterning, air exposure and cleaning followed by growth of a thick buffer layer) and the different results, compared to templating with Ga^+^, suggest that chemical effects may be crucial^31^. In contrast, we find here that Si^2+^ and Si^+^ ions form suitable nucleation sites for the assembly of pure Ge quantum dots in a process that appears similar to Ga^+^ ion templating. However, the ion dose required is larger and we find that it is associated with substantial amorphization of the implanted area. Recovery of this damage is important if Si ion templating is to be used to fabricate structures with controlled electronic properties. We find that the damage can indeed be recovered by heating to temperatures that are consistent with the formation conditions of Ge quantum dots by CVD. The recrystallization leaves residual line defects around the perimeter of the implanted area. We discuss the mechanism of this defect formation and propose that this process can be extended to produce complex patterns of defects, leading to another potential application where Si ion FIB is used to modify a substrate with local defects. Overall, we propose that the creation of both Ge quantum dots and defects arranged in arbitrary patterns is possible without associated chemical changes in the substrate. More generally, we expect FIB with Si ions, or mass-separated FIB with other species, to become an increasingly important tool in nanoscale fabrication.

## Results and Discussion

### Ge Quantum Dot Growth on Si^2+^ Templated Si

**Figure 1:**
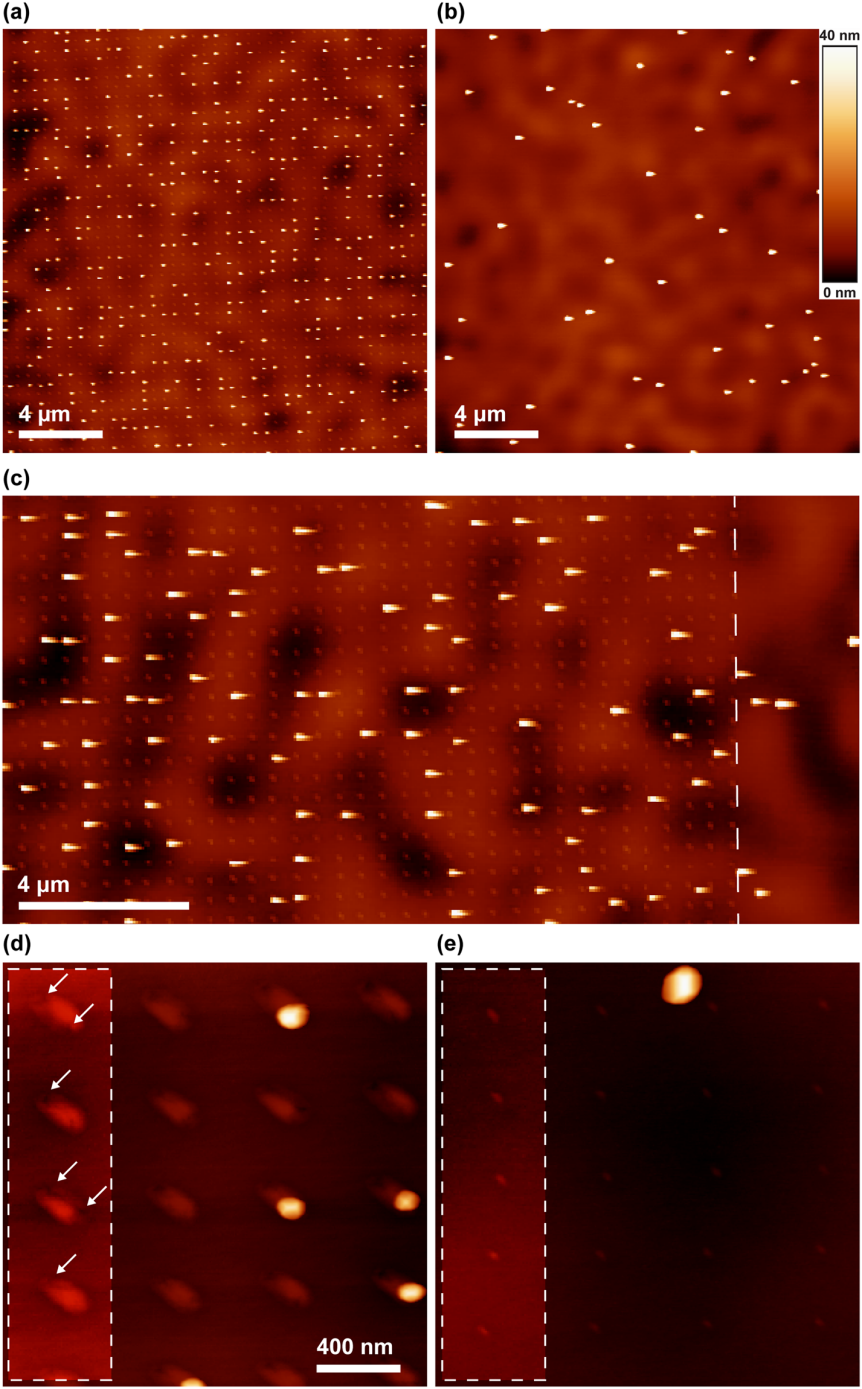
(**a**) Atomic force microscopy (AFM) image of Ge quantum dots grown on Si(100) patterned with 60 keV Si^2+^ ions at 1 × 10^6^ ions per spot in a square array of spots (500 nm separation). The implanted spots show up as shallow surface bumps and Ge quantum dots as the brighter spots. (**b**) AFM image of an area of the sample that did not undergo implantation. (**c**) Image acquired near the edge of the implanted region (indicated by the dotted line) showing the transition in quantum dot growth density. The shapes are distorted due to the speed of the scan. (**d**,**e**) Higher magnification images of two areas implanted with doses of 1 × 10^6^ and 1 × 10^5^ ions per spot respectively followed by Ge quantum dot growth. In (**d**), shallow bumps and pits (white arrows) are visible at implant sites. In (**e**) the bumps are smaller than in (**d**), no pits are visible and there is no correspondence between quantum dots and the implantation pattern. In both images the brightness and contrast in the dotted rectangle are adjusted to accentuate the features. We attribute the asymmetric shape of the surface bumps to residual astigmatism in the Si^2+^ ion beam.

In Fig. 1, we show atomic force microscopy images of Ge quantum dots patterned by using Si^2+^ FIB implantation on silicon-on-insulator (SOI) wafers. Details of the Si^2+^ ion generation are provided in the Methods. First, we compare the Ge quantum dot growth in the area templated with ions, Fig. 1(a), with an area of sample that did not undergo Si^2+^ implantation, Fig. 1(b). Alignment of the Ge quantum dots in the templated area is clearly visible. About 25% of the implant sites in Fig. 1(a) have an associated quantum dot. The overall quantum dot density in the templated area is higher (~1 µm^−2^) when compared to the area that was not implanted (~0.1 µm^−2^). Figure 1(c) shows that this density change occurs immediately at the edge of the implanted region. The quantum dots in the templated area are also smaller, ~150–200 nm across and ~40 nm in height, compared to ~250–400 nm across and ~60 nm in height in the area without irradiation (see Supplementary Figure 1). It is interesting to note the general absence of quantum dots between the implanted spots, an important requirement when patterning functional arrays of nanostructures. Figure 1(d,e) compare higher magnification images of areas implanted with two doses, 1 × 10^6^ ions and 1 × 10^5^ Si ions per spot. At the lower dose, there is no correspondence between the Ge quantum dot growth and the implanted array. The threshold dose required to induce patterning with Si^2+^ under these conditions thus appears to be in the 10^5^ ions per spot range, more than an order of magnitude higher than that reported for Ga^+^ ions (6 × 10^3^ ions per spot^10^).

To understand the mechanism for the patterning, we consider a model developed to explain how Ga^+^ implantation patterns Ge quantum dots^11^. Ga^+^ focused ion implantation into Si has two effects: it amorphizes the substrate and removes material from each implant site by sputtering. Amorphization leads to substrate swelling and a bump becomes visible on the surface at low doses, whereas sputtering eventually creates a surface crater at higher doses. The final surface morphology, due to these two competing effects, is a function of the ion mass, energy and dose, and the target material^32,33^. On the other hand, the annealing that occurs during heating under vacuum to the Ge growth temperature causes surface smoothing and recrystallization. Recrystallization takes place from the periphery of the implanted area inwards and the sputtered crater transforms into a few nanometer-sized surface pit surrounded by flat surface. It is the pit formed through sputtering plus annealing that acts as a nucleation site for a Ge island^11^, rather than other effects of Ga^+^ patterning such as the amorphization or any chemical changes.

**Figure 2:**
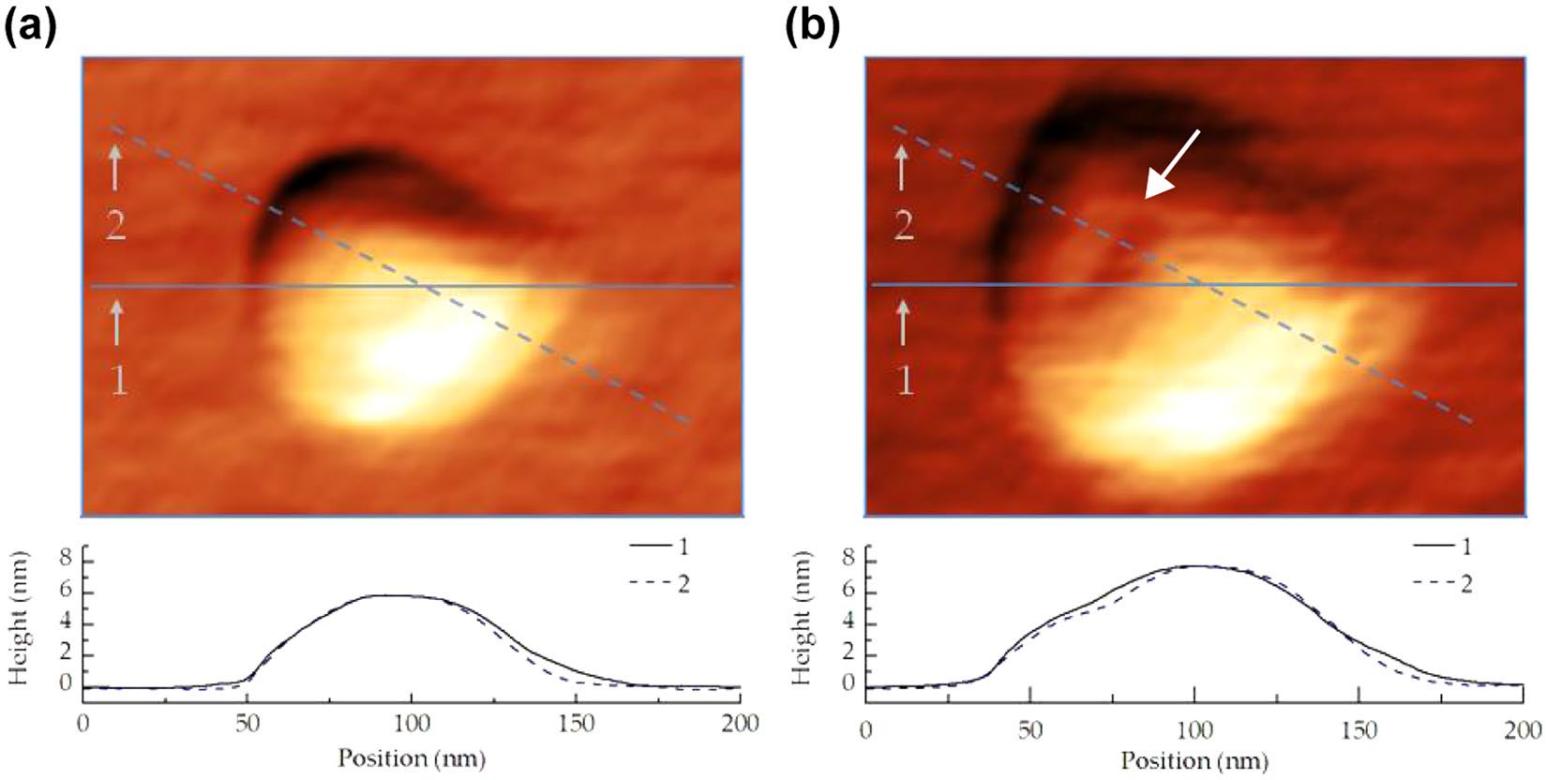
AFM images of a Si wafer that was implanted with 60 keV Si^2+^ ions at (**a**) 2 × 10^5^ ions per spot and (**b**) 6 × 10^5^ ions per spot and the line profiles corresponding to lines 1 and 2 in the respective images. Note the development of a small surface crater in (**b**) denoted by the white arrow. Adapted from ref.^32^.

While implantation with Si ions also creates surface bumps and craters in the same manner as Ga^34^, a key difference between Si and Ga is the sputter yield. The sputter yield is 2.3 Si atoms per ion for 30 keV Ga^+^ but only 1.2 Si atoms per ion for 60 keV Si^2+^, according to SRIM calculations^35^. If we also include the implanted Si^2+^, only a net 0.2 Si atom is removed per ion. To understand the effect of this difference in sputter yield, we examined how the surface morphology evolves as a function of dose by imaging the Si-implanted surface with AFM directly after implantation (Fig. 2 and Supplementary Figure 2). Substrate swelling dominates at low doses due to the amorphization caused by the implantation. Swelling increases with dose, and depressed features within the surface bump also become visible at doses of 6 × 10^5^ ions per spot. This behavior is consistent with the amorphization and sputtering effects seen during Ga^+^ implantation, although at higher dose. After Ge growth, these features remain visible in AFM. Figure 1(e) shows shallow bumps (~1 nm in height) at a dose (1 × 10^5^ ions per spot) that does not cause Ge patterning, and Fig. 1(d) shows larger bumps (~4 nm in height) with shallow pits (white arrows) at a dose (1 × 10^6^ ions per spot) that does pattern Ge. The features show some variation, for example two shallow pits instead of one can be seen in some implant sites. Although these datasets are obtained after air exposure, which may change the surface profile due to the increased oxidation of amorphized silicon^12^, the observations (especially the presence of the shallow pits) suggest that sputtering plus annealing creates surface pits at doses in the range (0.6–1.0 × 10^6^ ions per spot) at which patterning occurs, in agreement with the Ga^+^ model^11^. The higher dose required for patterning compared to Ga^+^ can therefore be understood as a consequence of the lower sputter yield of Si^2+^ ions. The higher required dose may also be related to the size of the irradiated spot. The ion optics in the mass filtered column produce a larger spot size for the Si^2+^ ion beam than does a typical Ga^+^ FIB tool (the manufacturer’s resolution test gives 62 nm versus 14 nm^30^). Qualitatively it may not be surprising that sputtering from this 20 × larger area followed by annealing is less efficient at generating the pit required for Ge nucleation. Indeed, multiple pits are often seen in Si^2+^ implanted spots (Fig. 1(d)). To test this interpretation, we show in Supplementary Figure 3 that Si^+^ ions, with sputter yield similar to Si^2+^ at 1.5, also enable Ge quantum dot patterning and require a much higher dose than Ga^+^: the dose is in fact somewhat higher than for Si^2+^.

The ~10^3^ × larger dose that Si^2+^ requires compared to Ga^+^ has a consequence for the amorphization that is necessarily associated with the patterning dose. According to SRIM calculations^35^, each 60 keV Si^2+^ ion causes 1400 atomic displacements whereas each 30 keV Ga^+^ ion causes 800 displacements. The range for the Si^2+^ ion in Si is about three times longer than the Ga^+^ ion (85 nm versus 27 nm). Therefore, although Ga^+^ and Si^2+^ patterning both cause collateral damage to the substrate, a deeper and broader volume is damaged by the Si^2+^ dose required for patterning. The recovery of this extensive damage creates opportunities for patterning that will be discussed below.

In spite of the higher dose required for patterning with Si^2+^ compared to Ga^+^, the above results are promising in demonstrating that patterning can be achieved using Si rather than a chemically different species. The results also support the interpretation of pit formation controlling nucleation rather than chemical effects. Hence, the formation of arrays of quantum dots can be achieved separately from the control of chemical composition in the patterned structure. However, the low occupancy of the patterned sites indicates that our control over the surface pit formation is not sufficiently precise and further systematic examination of deposition parameters will be required to improve the fraction of sites that are occupied by Ge quantum dots.

### Recovery of FIB Damage in the Si Substrate

**Figure 3:**
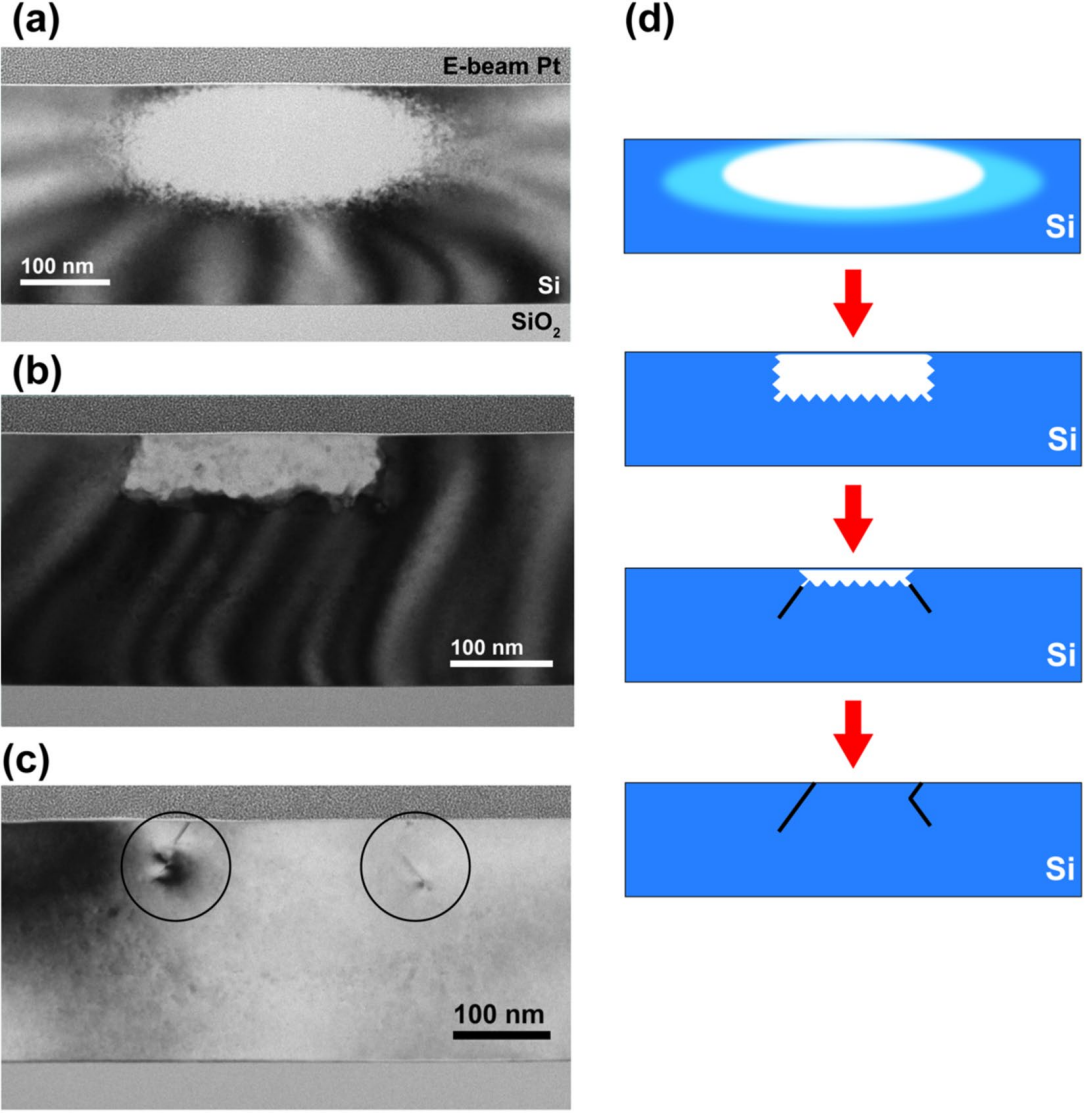
Bright field transmission electron microscopy (TEM) images obtained along the [011] axis from samples in which Si^2+^ was implanted in a pattern of lines along [011], each made up of 50 nm steps with ~1 × 10^5^ ions implanted per step. The dose is thus 2 × 10^3^ ions per nm. Cross sections were cut after (**a**) implantation, and after annealing at (**b**) 500 °C and (**c**) at 600 °C for 30 minutes. As-implanted (**a**), the amorphized area is ~370 nm wide and 140 nm deep. After (**b**) annealing at 500 °C, the amorphous area reduces to ~245 nm wide and 63 nm deep. After (**c**) annealing at 600 °C, recrystallization occurs but leaves a pair of residual defects, highlighted with black circles. The defect on the left makes an angle of ~55° with the surface, whereas the defect on the right has a kink with an angle of ~100°, consistent with the angle between {111} facets. In (**c**), the surface pit expected for these implant/anneal conditions would be too shallow to be visible. (**d**) A schematic of recrystallization via multi-directional solid phase epitaxial regrowth where the growth fronts form {111} nanofacets. Fully amorphous areas are shown in white and crystalline Si in blue. The transition area in the first panel indicates a partially amorphized region.

As mentioned above, compared to Ga^+^, the higher dose required for Si^2+^ patterning and the longer implantation range of Si^2+^ means that greater amorphization of the substrate^36^ is expected during implantation. The heat treatment that is part of the Ge quantum dot growth needs to recover this damage if we are to optimize the electronic properties of the resulting nanostructures. Hence, we use transmission electron microscopy (TEM) to characterize the recrystallization of Si implanted with focused Si^2+^ ions at temperatures up to 600 °C, the temperature used in our experiments for Ge growth. Figure 3 shows the recrystallization of Si implanted with lines of Si^2+^ ions written parallel to the [011] direction. Writing lines rather than spots made image interpretation in a cross-section more straightforward since the electron beam direction is parallel to the lines (although lines do not appear to be useful in inducing preferential nucleation of Ge quantum dots, presumably because they do not generate well-defined pits or other surface features on the surface). *Ex situ* heating was used, in which cleaved portions of the as-implanted wafer were heated at specific temperatures and then cut into thin sections using standard FIB sample preparation techniques.

**Figure 4:**
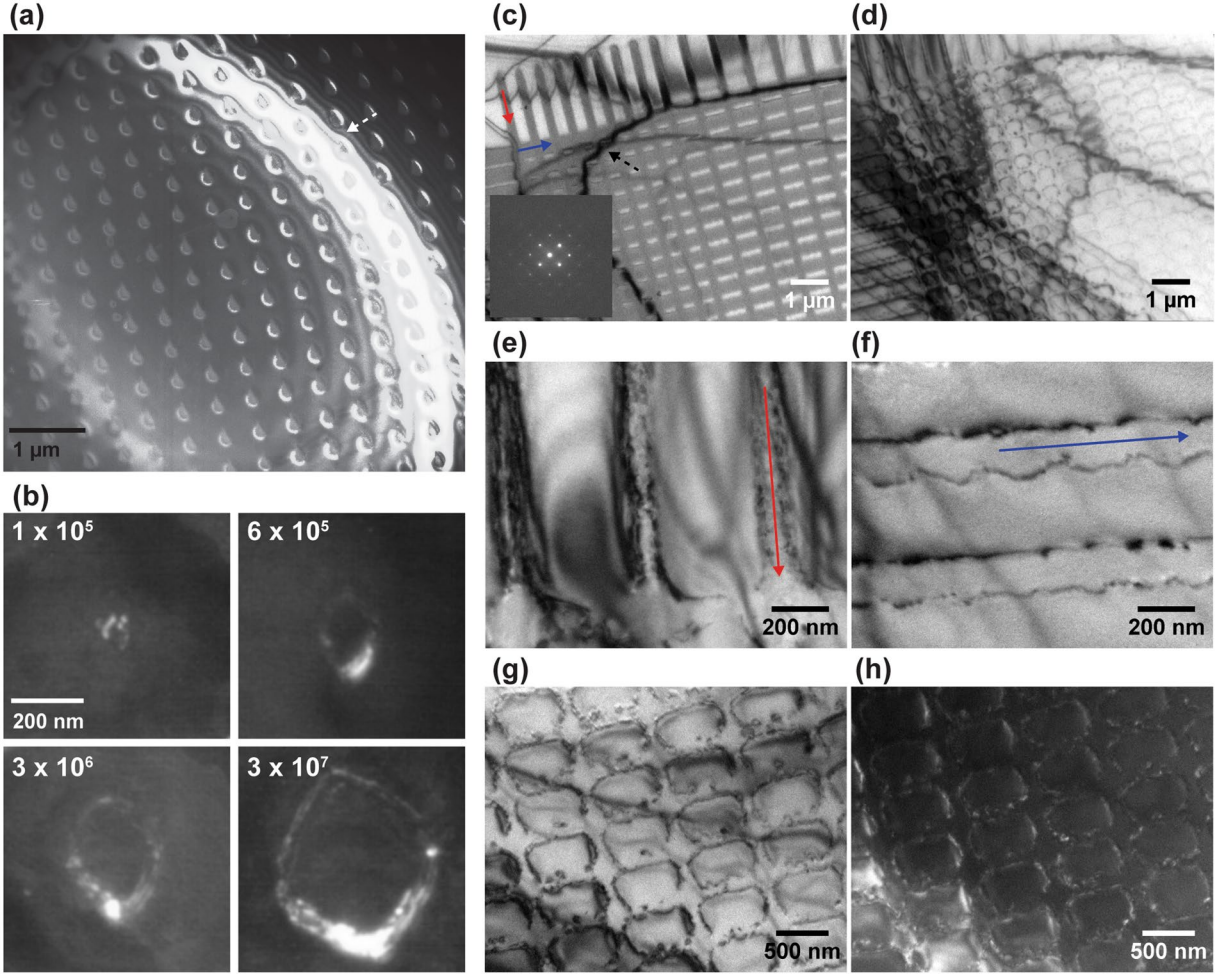
(**a**) Dark field TEM of an array of amorphous spots (5 × 10^4^ ions per spot) formed by 60 keV Si^2+^ ion implantation into a 220 nm thick Si(001) membrane. The bright bands (white dotted arrow) in the image are bend contours caused by membrane buckling. (**b**) Dark field TEM of the residual defects formed in the substrate by implantation at the doses shown followed by anneal *in situ* at 600 °C. (**c**) Bright field TEM of amorphized lines generated by implanting a row of closely spaced spots. The dose per spot is 1 × 10^5^ ions and the distance between spots is 50 nm. Note the different width of the two sets of lines, ~210 nm for top to bottom lines (red arrow) and ~350 nm for left to right lines (blue arrow), attributed to astigmatism of the FIB spot. The lines were patterned without specifying an orientation with respect to the substrate crystal; inset is the Si diffraction pattern. The dark bands (black dotted arrow) are bend contours caused by membrane buckling. (**d–f**) Bright field images recorded after heating *in situ* to 600 °C showing recrystallization and residual line defects that track the original pattern. (**d**) shows the area in (**c**) and (**e**,**f**) are higher magnification images showing the defects along each set of lines. (**g**,**h**) Bright and dark field images of an area near the center of the grid pattern. The bright field image is taken close to the [001] zone axis and the dark field image with a {400} diffraction spot.

Figure 3(a) shows the as-implanted wafer. The amorphous region is elliptical in cross section with lateral dimension ~370 nm and a depth of ~140 nm. The depth is in approximate agreement with the projected range predicted by SRIM^35^ (projected range ~90 nm, straggle ~ 30 nm). The lateral dimension is larger than we have observed previously from spot implantations^30^ (~170 nm at 8 × 10^5^ ions per spot), but this may be explained by less well optimized ion beam parameters in this particular experiment or the tails in the ion distribution within the beam. Around this central amorphous region, there appears to be a shell of partially amorphized Si, evidenced by the crystal contrast with superimposed speckles from individual defects. Heating to 500 °C recrystallizes the shell and leaves an amorphous region bounded by relatively flat sidewalls (Fig. 3(b)). The depth of this region is approximately the depth at which the initial amorphous region is widest. Its boundaries show small facets; small crystallites (darker areas) can be seen within the amorphous region. Heating further to the Ge growth temperature leads to a second stage of microstructural evolution in which the central amorphous area fully recrystallizes, but defects remain at the periphery of the recrystallized region (Fig. 3(c)). These defects appear from the image contrast to be dislocation loops (also see Fig. 4) that extend from the surface down to a depth roughly consistent with the lower amorphous-crystalline interface in Fig. 3(b). The central recovered regions are relatively defect free, in agreement with the damage recovery measured with Raman spectroscopy on annealing Si substrates rendered amorphous by a focused Si^2+^ ion beam^36,37^. These data confirm that Si^2+^ implantation at doses relevant to quantum dot patterning, followed by heating to the temperature required for nanostructure growth, can result in defect-free regions beneath the grown nanostructure but with defects around the perimeter of the implanted area.

To follow the recovery process and the formation of the defects in more detail, and to compare the recovery processes for spots and lines, annealing was followed *in situ* in a plan view (top-down) geometry using samples implanted with Si^2+^ ions in patterns containing both lines and spots. Imaging was carried out in electron-transparent windows that were created by etching the handle wafer and buried oxide layer to leave a Si membrane (see Materials and Methods). After patterning the membrane region with FIB, the samples were then heated *in situ* in the TEM.

The images in Fig. 4(a,b) show recovery of the damage produced by Si^2+^ implantation of spots. The recovery process in implanted spots is consistent with our previous studies of implantation with Si^2+^ ions^30^. Annealing an implanted spot leaves behind a continuous defect loop marking the boundary of the amorphized region and the size of this loop increases with dose (Fig. 4(b)). The images in Fig. 4(c–h) and Supplementary Figure 4 show recovery of the damage produced by implantation of lines and grids. The observations are consistent with the two-stage process described in Fig. 3. The amorphous areas start to reduce in size at temperatures around 300 °C to 400 °C (see Supplementary Figure 4 for additional images as a function of temperature), followed by recrystallization of the central region at around 600 °C, leaving defects at the periphery of the lines. The substrate recrystallizes completely within ~60 sec of reaching 600 °C. In Fig. 4(e,f), we compare the effects of annealing on lines drawn in the two perpendicular orientations. We note that the lines have identical dose per length but different initial widths due to asymmetry in the ion beam profile, hence a different effective dose per unit area. The narrower lines in Fig. 4(e) reduce from ~210 nm to ~180 nm, but further annealing leaves defects in the central region. The wider lines in Fig. 4(f) reduce from ~350 to ~250 nm in width during the first stage of annealing, then fully recrystallize to leave almost no defects in the central region and a pair of dislocations running along the periphery. In Fig. 4(g,h), we use bright and dark field imaging to show that the residual defects outline the holes in the grid pattern. The differences in geometry between spots, lines and the grid do not appear to affect the fundamental recovery process. Initial spot diameters and line widths are of the order of a few hundred nm, whereas in the recrystallization process in Figs 3 and 4 the residual defects form within 50 nm of the edges of the amorphized region.

The formation mechanism of these residual defects can be understood by considering the many prior studies of Si recrystallization under various circumstances. Residual defects are known to form during annealing of Si that is patterned with focused beams of Ga^+ ^^18,38^, B^+ ^^39^ and P^+ ^^39^ ions. But in our case, since no foreign ions are implanted, the defect formation process may be more analogous to the formation of mask-edge defects^39,40^ via multi-directional solid phase epitaxial regrowth (SPER)^41^, which can occur at the ~500–600 °C temperature relevant here^42^. In multidirectional SPER, lateral and vertical amorphous-to-crystalline growth fronts impinge and defects (known as clamshell or zipper defects) are generated because of mismatch at the junctions between growth fronts^43^. At the atomic level, recrystallization in the < 001 > and < 110 > growth directions occurs via the formation and filling in of {111} nanofacets on the interfaces^39,40^. When the (001) growth front moving upwards meets the {110} growth fronts moving inwards, mis-registry on the {111} planes is possible. Figure 3(d) shows a schematic of this defect formation mechanism. If the lateral fronts meet before the vertical front reaches the surface, the remaining amorphous area will be bounded by {111} facets and re-growth on the {111} surfaces is expected to be highly defective^42^. In this picture it is straightforward to explain, from the geometry of the impinging recrystallization fronts, why narrower FIB-patterned lines appear to have more defects after annealing. Furthermore, the angle between the defects and the surface in Fig. 3(c) is consistent with the defects being on {111} planes.

So far, we have shown that the patterning of Ge quantum dots by Si implantation appears to involve several distinct phenomena. The sputtering of Si to form the pits that act as nucleation sites also involves amorphization of a surrounding volume of the substrate. Recrystallization leads to the formation of defects around the periphery of the amorphized region. It is currently unclear if these defects correlate with the surface pits. On the other hand, the strain due to these defects may exert some control over quantum dot nucleation. The distorted bend contours seen in Fig. 4 indicate that the implantation introduces local strain to the substrate that remains after the annealing and formation of the defect structures. Highly localized strain fields have been shown to influence the nucleation of Ge quantum dots on Si^44,45^. Data shown in Supplementary Figure 4 suggests that we should be able to grow Ge quantum dots as close as 100 nm from each other with proper optimization of the growth parameters, but we may not be able to pack the implanted spots much closer, given the implanted lines do not control quantum dot nucleation. Alternatively, we can consider the strategy of using a spot that is deliberately made astigmatic to create two or more pits with fixed separation from each other.

**Figure 5:**
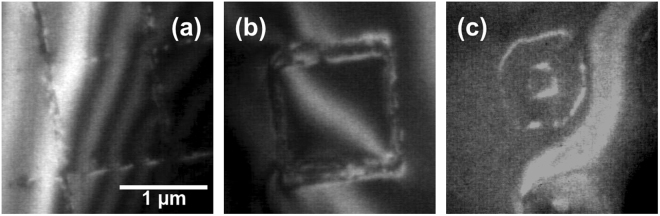
Dark field images of defect structures formed after annealing different implanted patterns. (**a**) a square grid, (**b**) a square and (**c**) a donut shaped structure formed with a defocused beam. A (111) reflection is used for imaging. Si^2+^ implantation conditions were 2 × 10^3^ ions per nm (~6–7 pA beam current, similar to Fig. 3).

It is also clear from Fig. 4 that FIB patterning can be a novel way to write defect structures into Si directly. The ability to program the geometry of defects generated by the amorphization plus annealing procedure creates additional opportunities for fabricating nanostructured arrays. We demonstrate this in Fig. 5 where we optimized the implantation conditions to form narrow defect lines that go around near-perfect Si (Fig. 5(a,b)) or defocused the beam to form donuts that are outlined by defects (Fig. 5(c)). The ability to write and control the location of these defect structures may be used to create defects that act as subsequent segregation sites for metal atoms^46^ and potentially form unique conducting nanostructures.

## Conclusions

We have shown that arrays of self-assembled Ge quantum dots can be patterned on a planar Si surface that has been modified with a Si^2+^ focused ion beam. Quantum dot patterning requires doses of the order of 10^6^ Si^2+^ ions per spot, followed by annealing at the Ge growth temperature to recrystallize the implanted region. While the growth conditions remain to be further optimized, it is clear that the presence of Ga, as in conventional FIB patterning, is not necessary for nucleation control. Instead, the formation of a pit by selection of appropriate sputtering and anneal conditions appears to be the determining factor for directing the positions at which Ge quantum dots nucleate. Hence, we can decouple the patterning of a substrate from the chemical composition of the substrate, for example the doping with Ga which is unavoidable if Ga^+^ FIB is used. The ion dose required for patterning with Si^2+^ is, however, higher than the dose required for nucleation control with Ga^+^. This can be explained by the difference in energy deposited into the sample per ion. At this dose, the Si^2+^ beam permits a second type of nanoscale patterning: it can be used to write defect structures into Si in patterns around the originally implanted area. We suggest that these defects are formed during solid phase epitaxial regrowth of the localized amorphous region. We conclude that MS-FIB with Si^2+^ ions has the potential to provide a palette of patterning approaches. It is promising for the formation of arbitrarily complex patterns of Ge quantum dots without associated changes in doping, which may become an important general tool in quantum dot-based device fabrication. We anticipate various future applications of this powerful tool to create unique types of nanostructures while also controlling the chemistry of the material.

## Materials and Methods

### Si^2+^ Ion Implantation and Ge Quantum Dot Growth

To generate a focused beam of Si^2+^ ions, we used a 30 kV MS-FIB Canion 31 + column (Orsay Physics, Fuveau, France). This column is installed in a custom-built ultra-high vacuum (UHV) chamber^30^ that also has capabilities for heating and for gas delivery for CVD, as well as a built in scanning electron microscope (SEM) that allows imaging before and after ion implantation or nanostructure growth.

The samples were made from SOI wafers with a 220 nm thick single crystal Si(001) device layer on a 2 µm SiO_2_ layer on 750 µm Si^11^. 3 mm × 2 mm samples were diced from the wafer and then mechanically thinned to 200 µm. The samples were then loaded into a sample carrier capable of resistive heating in the UHV system. Each sample was briefly flashed at above 1000 °C in a UHV preparation chamber before transfer into the FIB chamber for templating.

The ions were generated from a AuSi liquid metal ion source, which emits a spectrum of monoatomic ions and polyatomic ions that may be singly or doubly charged^47^. These ion species were then separated according to their mass/charge in the Wien filter and the desired species selected with a slit. Note that the doubly-charged Si^2+^ ions are effectively accelerated to 60 keV while Si^+^ is accelerated to 30 keV.

Si^2+^ ions were delivered as an array of single shot spots at doses ranging from 2 × 10^4^ to 6 × 10^7^ ions per spot. The Si^2+^ beam current used ranged from 6 to 36 pA and the total implantation time ranged from 0.5 ms to 50 ms per spot. The ion beam parameters were optimized at the start of each experiment. The Si^+^ ion beam proved harder to optimize due to its lower intensity compared to Si^2+^. Furthermore, to avoid prior implantation of the area used for growth experiments, the sample was moved several tens of micrometers away from the area used for alignment, which presumably led to errors in alignment conditions for our implanted spots. In principle, the presence of the Si isotopes (^29^Si, ^30^Si) can also distort the shape of the implanted spot. In this case, we used a filter setting that displaces the isotopes away from ^28^Si by a few hundred nm to several micrometers^32^. At the highest implantation doses, we could occasionally see a signature of these separated spots as a small change in contrast of the Si membrane.

After implantation and without breaking vacuum, Ge quantum dots were grown onto the sample. Each sample was resistively heated to the growth temperature of ~600 °C, as calibrated using a pyrometer. Ge was then grown by introducing digermane (diluted in 80% helium) as a source gas into the chamber via a leak valve to a pressure of 1.5 × 10^−6^ Torr for 5 minutes. Post-growth, the sample were removed from the vacuum system and examined in air using AFM with a Digital Instruments Dimension 5000.

### ***In Situ*** TEM of FIB Damage Recovery

For *in situ* TEM experiments, a small electron-transparent region at the center of each sample was formed prior to implantation by buffered oxide etching from the back side up to the device layer to leave a silicon membrane.

The *in situ* heating experiments were carried out in two different TEMs. One was a 300 kV Hitachi H-9000 TEM with TV-rate video capture capabilities and a home built resistive heating stage, and the other was a 200 kV JEOL 2011 LaB_6_ TEM using a double tilt heating holder (Gatan, Inc., Pleasanton, CA). For the H-9000, the temperature reached by the substrate at each heating current used was calibrated using an infrared pyrometer after the annealing experiment. For the JEOL 2011, the sample temperature was measured from a thermocouple attached to the heating furnace. In both cases, the sample temperature was increased in incremental steps every few minutes and the sample imaged at each temperature.

### Data availability statement

The datasets generated and analysed during the current study are available from the corresponding author upon reasonable request.

## Electronic supplementary material

Supplementary Material
