# Supplementary material for: Directed Self-Assembly of Ge Quantum Dots Using Focused Si2+ Ion Beam Patterning
Source: Sci Rep. 2018 Jun 19;8:9361. doi: 10.1038/s41598-018-27512-z (PMC6008470; doi:10.1038/s41598-018-27512-z)
Supplement: Supplementary file 1 — Supplementary Material [file 41598_2018_27512_MOESM1_ESM.docx]

**Electronic Supplementary Information**

Directed Self-Assembly of Ge Quantum Dots Using Focused Si^2+^ Ion Beam Patterning

See Wee Chee^1^, Martin Kammler^2^, Jeremy Graham^3^, Lynne Gignac^4^, Mark C. Reuter^4^, Robert Hull^1^, Frances M. Ross^*^

1. Department of Materials Science and Engineering, Rensselaer Polytechnic Institute, Troy, NY 12180.
2. Institut für Experimentelle Physik, Universität Duisburg-Essen, 47048 Duisburg, Germany.
3. Department of Materials Science and Engineering, University of Virginia, Charlottesville, VA 22904, USA.
4. IBM Research Division, T.J. Watson Research Center, Yorktown Heights, New York 10598, USA.

* Correspondence: [fmross@us.ibm.com](mailto:fmross@us.ibm.com)


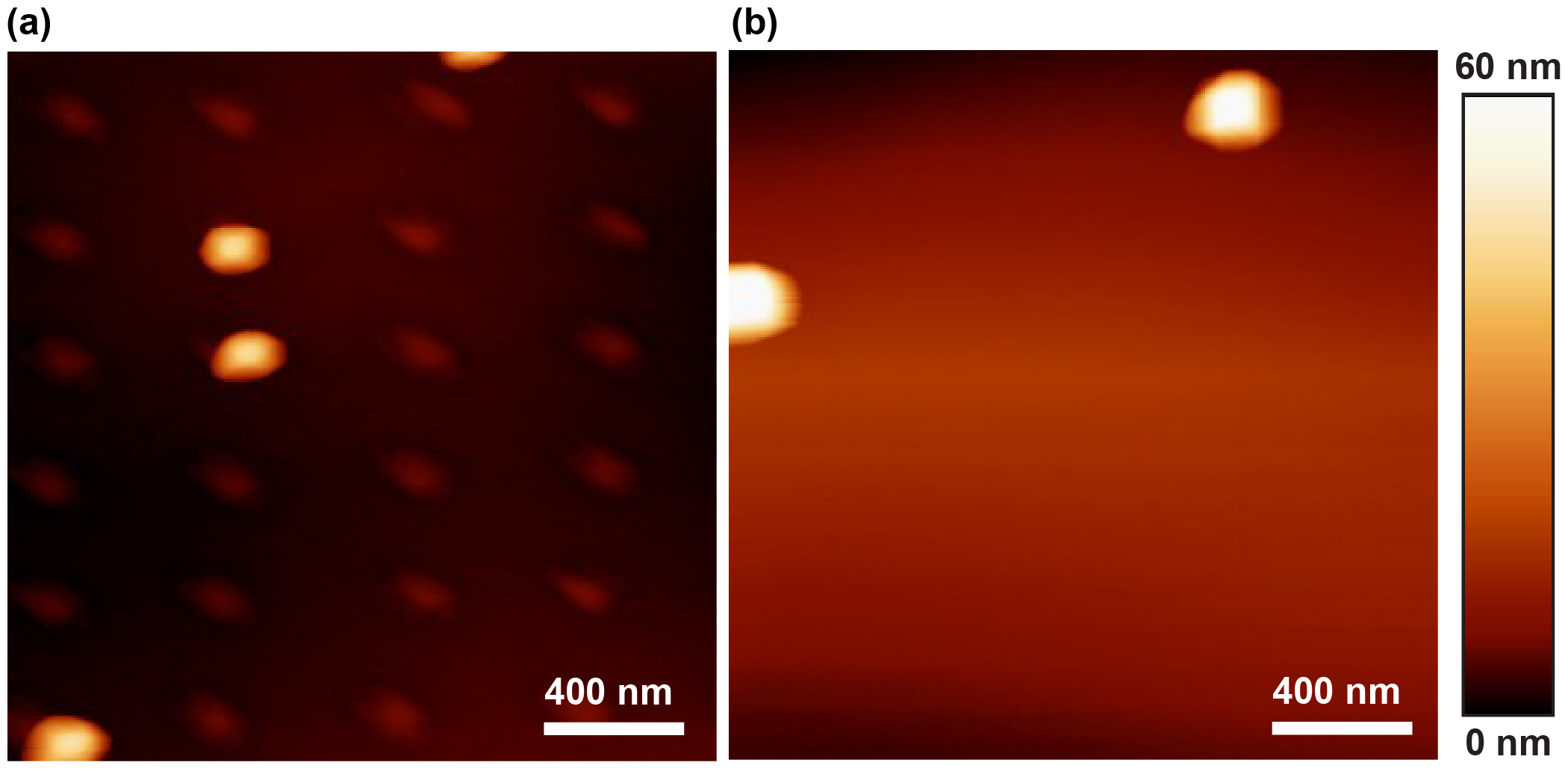


ESI Figure 1. AFM images from taken from (a) an area that is templated with 30kV Si^2+^ ions at 1 × 10^6^ ions per spot and (b) an area that is not ion irradiated.


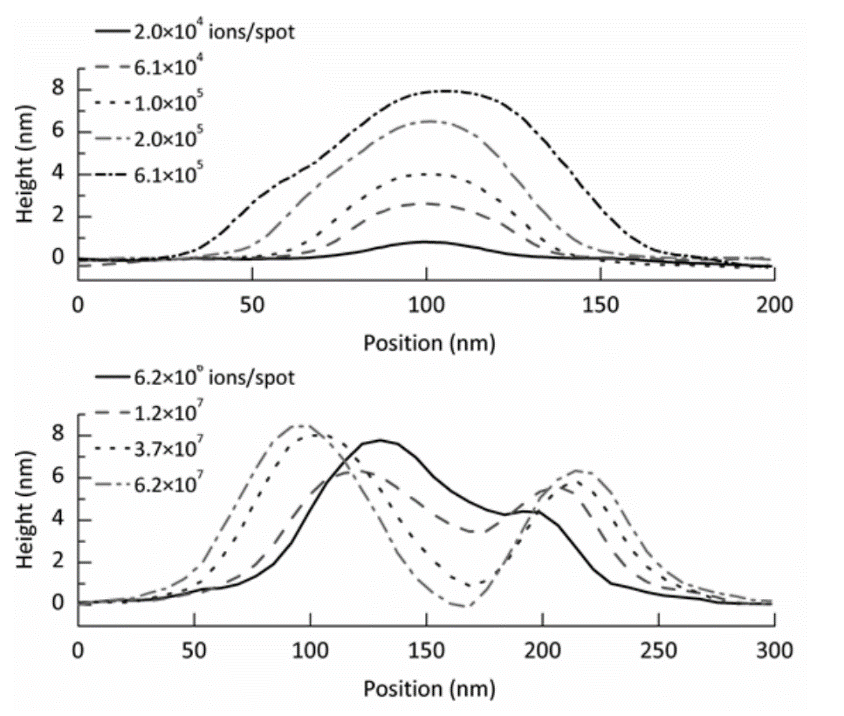
ESI Figure 2. Atomic force microscopy (AFM) line profiles of surface bumps on Si due to Si^2+^ ion implantation. Doses of 2 ×10^4^ to 6 ×10^7^ ions per spot are shown in the two sets of line profiles. Note the development of pits above 6 ×10^5^ ions per spot. A different but identical model Canion 31+ FIB column was used to perform these implantation experiments.


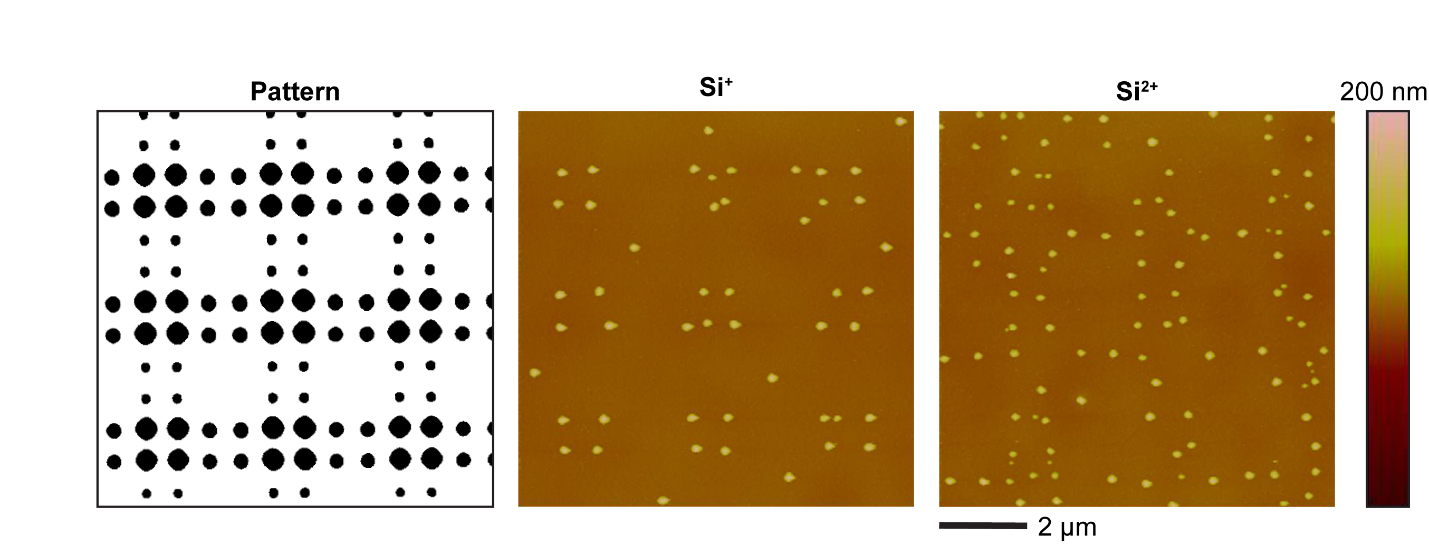


ESI Figure 3. Ge island growth on Si substrates patterned with Si^+^ and Si^2+^ ions. The left panel depicts the implantation pattern where the size of the dot indicates the ion dose. The doses used were 3 × 10^7^, 3 × 10^6^ and 7 × 10^5^ ions per spot. Both ions require high doses to cause patterning compared to Ga^+^: all three doses lead to patterning with Si^2+^, whereas only the highest dose led to patterning with Si^+^. This may reflect the less optimized spot size of Si^+^ compared to Si^2+^. Here, we also find that some implanted sites nucleated two quantum dots.


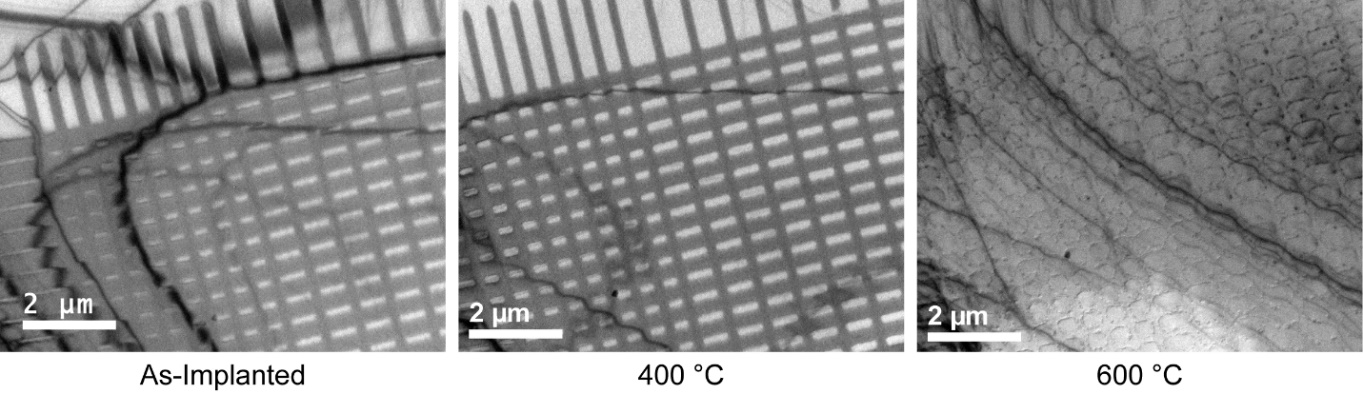


ESI Figure 4. TEM images of the grid pattern as the sample temperature was increased from room temperature to 600 °C. Note the reduced width of the amorphous lines at 400 °C and the recrystallization of the sample and formation of defect patterns at 600 °C.
